# Salvage of Exposed Breast Implant Using Capsular Flaps

**Published:** 2009-09-17

**Authors:** Francesco Gargano, Frank Ciminello, Silvio Podda, Giorgio De Santis

**Affiliations:** ^a^Miami Children's Hospital, Miami, FL, 33155; ^b^UMDNJ Plastic Surgery Department, Newark, NJ 07103; ^c^St Joseph's Childrens Hospital, Paterson, NJ 07503; ^d^Department of Plastic Surgery, University of Modena and Reggio Emilia, Modena 41100, Italy

## Abstract

**Objective:** Extrusion represents potential complications associated with the use of breast implants. Attempts to salvage the exposed implants are rarely successful when poor tissue coverage or radiotherpy is present and therefore removal of implant and wound healing are mandatory. In these refractory complicated cases the use of capsular flaps can represent a useful tool to save the implant and achieve definitive healing. **Methods:** Capsular flaps have been performed on 6 patients with implant extrusion and 11 patients with breast contour deformities over the last 6 years. The authors describe an innovative technique using capsular flaps which are harvested from thicker viable tissues and inset in multiple layers into the fistula tract to reinforce the breast envelope and prevent recurrence of implant extrusion. **Results:** Complete healing and implant salvage were achieved in all patients treated. No major complications occurred and only minor contour deformities, that regressed spontaneously after surgery, were observed for 2 weeks. **Conclusions:** Although capsular flaps have been previously described to correct breast shape deformities, no previous report has yet suggested its utility in breast implant salvage in case of extrusion. The authors advocate the use of capsular flap to save the exposed breast implant especially when poor tissue coverage is present and other surgical options to save the implant have already failed.

Breast implant extrusion represents a challenging problem that concerns the breast reconstructive surgeon. Implant exposure due to poor tissue coverage frequently obligates the surgeon to remove the breast implant and begin anew. In the authors' review of the literature, successful salvage of breast implants has been reported^1^–^3^ and guidelines for treatment have been recently proposed.^4^ Recommended treatment strategies include attempts to salvage the implant by antibiotic therapy, pulse lavage, capsulectomy, device exchange, primary closure, and/or flap coverage.^1^–^3^ Techniques of implant salvage have proven useful in cases with good tissues viability and in the absence of radiotherapy. However, in the face of implant exposure and poor soft tissue coverage, the outcome is often less optimal.^4^

Breast surgery with implants requires good tissue coverage, especially in cases in which adjunctive radiation therapy has been used. Wound complication, such as dehiscence, infection, and fistulas,^5^,^6^ and clinically significant capsular contracture have been well reported in irradiated breast tissue.^7^

The irregular surface of the implant, the location of the pocket, and the pressure exerted by the implant against the skin can also lead to implant extrusion.^8^

Capsular flaps have been first described by Gargano et al^9^,^10^ and later by other authors,^11^,^12^ with the original goal to correct postimplant breast wrinkling and contour deformities. To our knowledge, no previous reports have yet described the use of capsular flaps to save extruded breast implants. We postulate that capsular flaps could be extremely useful to reinforce the damaged breast envelope due to poor tissue coverage in patients who have undergone radiation therapy.

The authors outline an innovative technique using capsular flaps to salvage exposed breast implants.

## METHODS

Capsular flap procedures were performed on 6 patients with implant extrusion and 11 patients with breast contour deformities over the last 6 years. In all cases, preoperative planning defined the extent of the fistula and the deformity to be corrected. Parameters considered during the preoperative planning were skin quality, presence of infection, previous radiotherapy, and location of the defect. Capsular flaps were then designed to cover areas of wound separation and implant exposure. We operated on 5 patients with extrusion of the implant after breast reconstruction. In these cases, several attempts to save the device by fistula excision or implant removal with direct soft tissue suture over a new prosthesis all resulted in reexposure. The use of capsular flaps resulted in successful healing and implant salvage in all these cases. The sixth patient, a transsexual who during sex reassignment surgery had liquid silicone injections and breast implantation, was operated on only for cosmetic purpose. He developed recurrent fistulas resistant to traditional treatment on 1 breast. Capsular flaps were used to reinforce the breast envelope. A new implant was inserted and the outcome was successful.

## CASE REPORTS

### Patient 1

**Figure 1:**
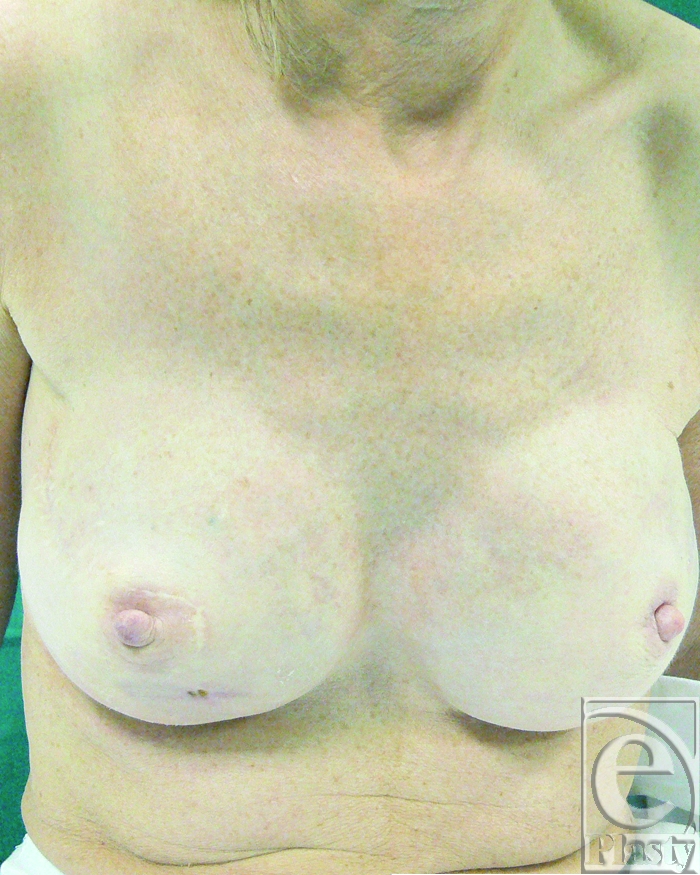
Preoperative view of the right breast with 2 fistulas at the inferior border of the nipple. The patient had 4 previous attempts of fistulas removal, implant exchange, and direct closure.

A 45-year-old woman underwent a bilateral nipple-sparing mastectomy and breast reconstruction with expanders and implants. The breasts were not irradiated in this case. After 1 month, the patient developed 2 fistulas located 3 cm below the right nipple, exposing the implant on the skin surface. Four previous surgical procedures were attempted to save the implant by direct excision, implant exchange, and direct wound closure. All attempts failed and implant extrusion recurred. The use of capsular flaps was proposed as an alternative treatment modality to breast implant removal (Fig 1).

**Figure 2:**
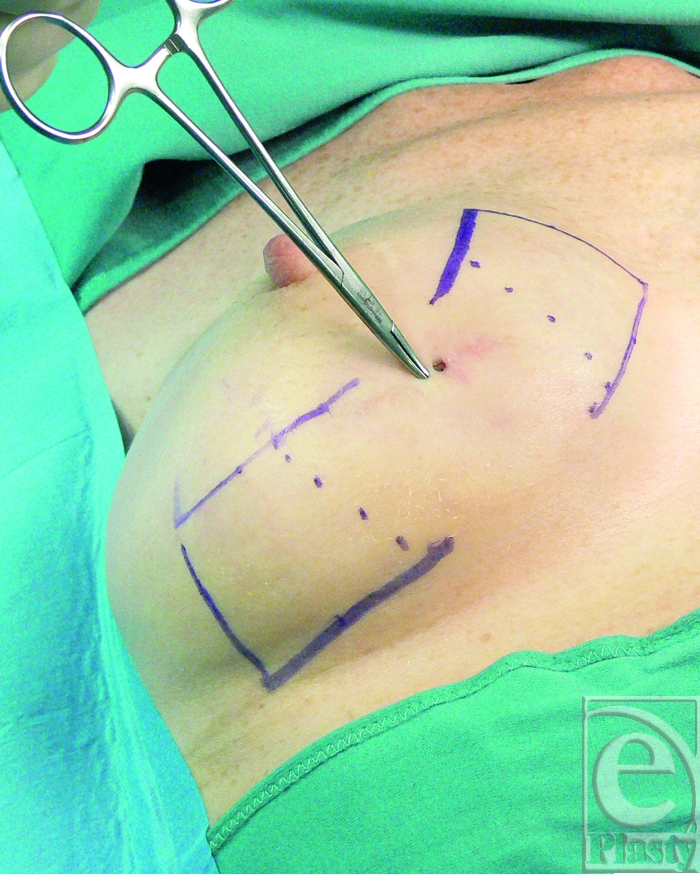
Intraoperative view of the location of the 2 fistulas and planning of the 2 capsular flaps that have been marked on the skin surface. The flaps are harvested in an area with viable tissues and their bases are dotted 4 cm away from the fistula site.

At the time of surgery, the 2 fistulas were outlined and the planned capsular flaps were drawn on the skin surface (Fig 2).

**Figure 3:**
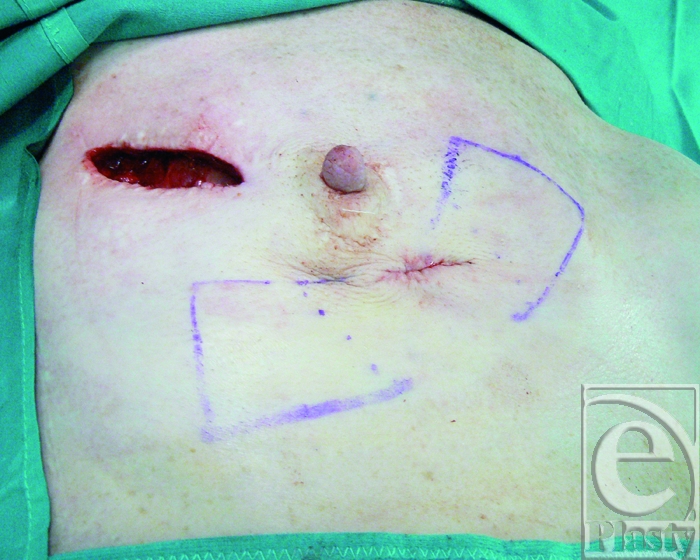
The fistulas are excised and sutured inside out. Skin incision is performed along the previous surgical scar and direct access to the capsular flaps donor site is achieved.

The previous surgical scar was chosen as skin access to the implant pocket. The fistulas were excised with the surrounding scar tissues (Fig 3).

**Figure 4:**
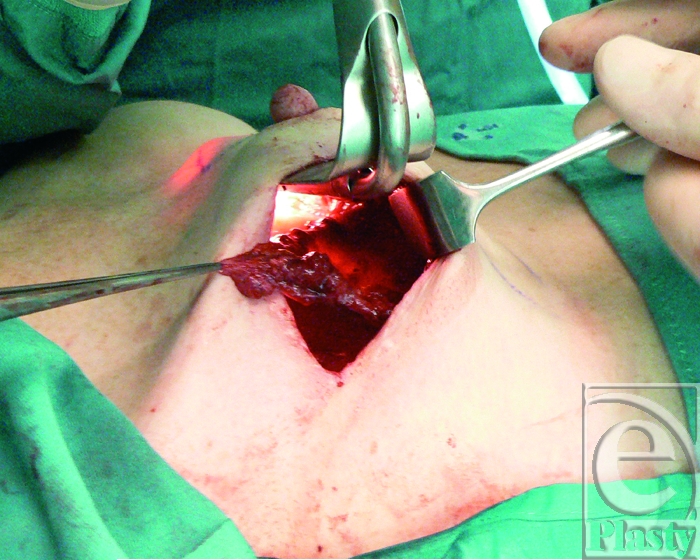
The capsular flaps are harvested from viable and thick tissues and then hinged on their base pivot point and inset in double layer over the fistula tract.

**Figure 5:**
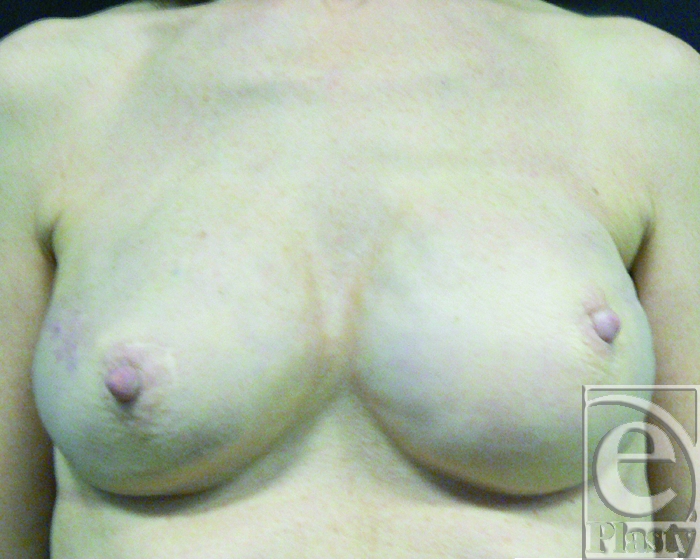
Eighteen months postoperative results showing good cosmetic appearance and the absence of implant extrusion recurrence.

Two inferiorly based capsular flaps were elevated and then folded over the defect. The defect was then closed primarily (Fig 4). Postoperative follow-up within 18 months showed no evidence of reexposure, with reasonable aesthetic result (Fig 5).

## Patient 2

**Figure 6:**
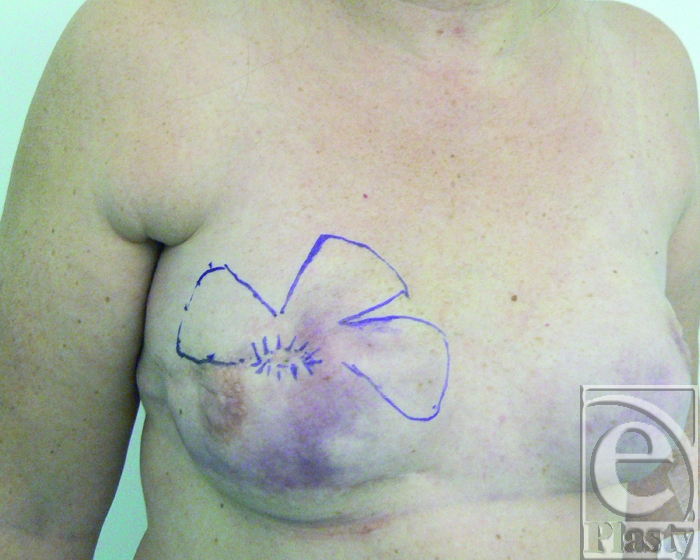
Preoperative view of a 57-year-old woman with soft tissue radiation injuries and breast implant extrusion. Three capsular flaps and lateral approach to the pocket were planned surgically. Flap donor site and skin incision are chosen according to the viability of the tissues.

A 54-year-old woman underwent right lumpectomy and radiotherapy. After local recurrence, bilateral nipple-sparing mastectomy and breast reconstruction with expanders and implants were performed. The right breast developed postradiation injury with subsequent exposure of the implant. Five attempts to save the right breast implant were unsuccessful and implant exposure recurred. Surgical options were explained to the patient and the use of capsular flaps was proposed as an implant salvage technique. Three inferiorly based capsular flaps were designed on the skin surface in areas with evidence of less radiation damage (Fig 6).

**Figure 7:**
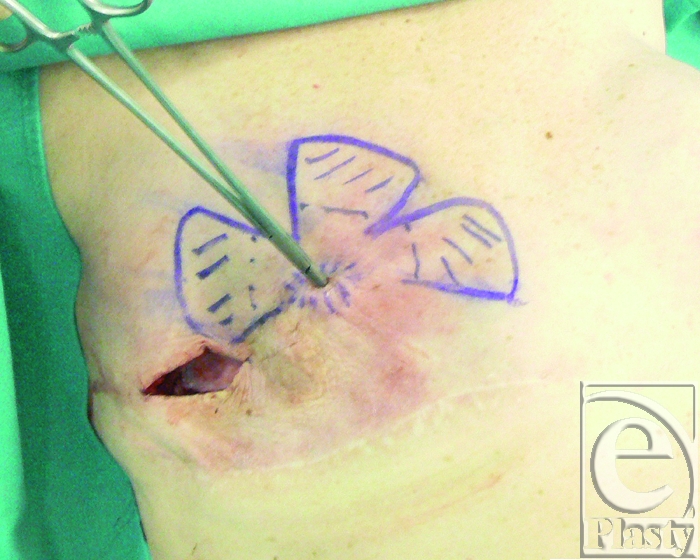
Intraoperative view of the same patient showing the skin access located on undamaged tissues, fistula tract identification, and extent of capsular flap harvesting.

The fistula track and all surrounding irradiated scar were excised. The capsular flaps were based inferiorly and included tissue outside the zone of irradiation (Fig 7).

**Figure 8:**
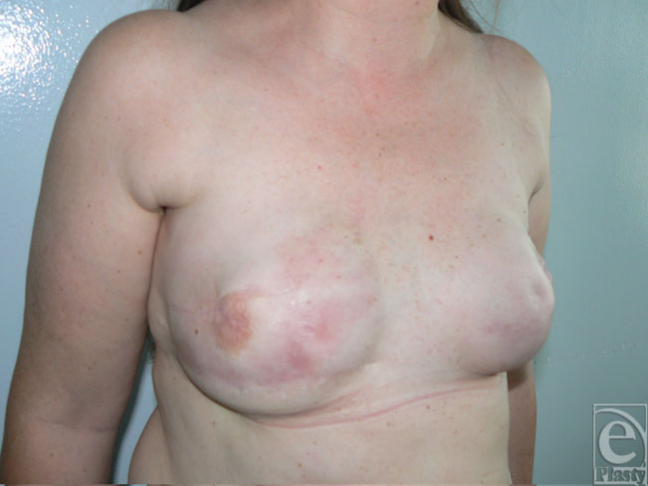
Twelve months postoperative follow-up showing complete healing and no recurrence of implant extrusion. Skin quality results to be improved with capsular flaps.

The 3 flaps were layered over the defect and skin was closed primarily. A 12-month postoperative follow-up showed no recurrence of implant exposure and improved aesthetic appearance of the soft tissues overlying the implant (Fig 8).

## Patient 3

**Figure 9:**
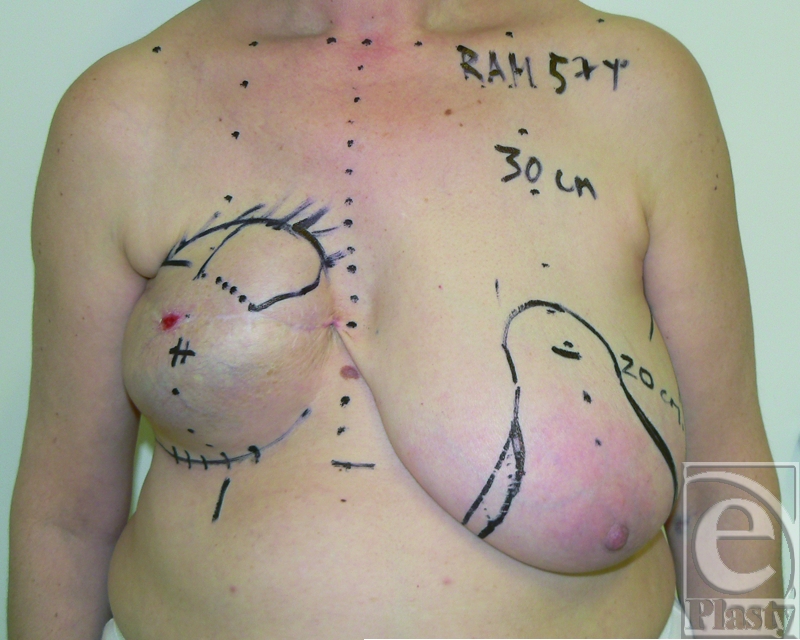
Preoperative view of fistula tract formation on extremely damaged soft tissues of the right breast: adequate expansion was achieved despite the risk of severe infection. Surgical planning considered was not to further damage the thin skin breast envelope and therefore an inframammary incision and posterior capsular flaps were chosen.

A 57-year-old woman underwent right mastectomy, followed by radiotherapy and breast reconstruction with expander. Extensive right breast radiation injuries occurred and a fistula tract developed over the infusion port of the tissue expander. Preoperative evaluation revealed thin and damaged skin coverage over the majority of the breast surface. Therefore, a submammary incision was planned to access the expander pocket in order to avoid this irradiated tissue. A single superiorly based capsular flap was harvested from the posterior capsule (Fig 9).

The expander was then removed and the fistula was excised. The capsular flap was inset in a single layer over the implant with pullout sutures to avoid traction on the irradiated skin. A vertical scar-reduction mammoplasty was performed on the left breast to achieve symmetry.

**Figure 10:**
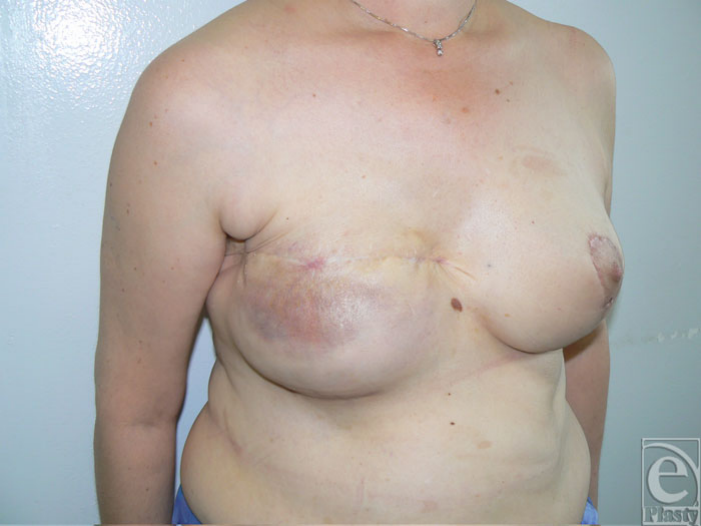
Postoperative results showing improved skin quality and salvage of the implant.

Postoperative results revealed complete healing of the irradiated soft tissues and salvage of the definitive implant (Fig 10).

## Patient 4

**Figure 11:**
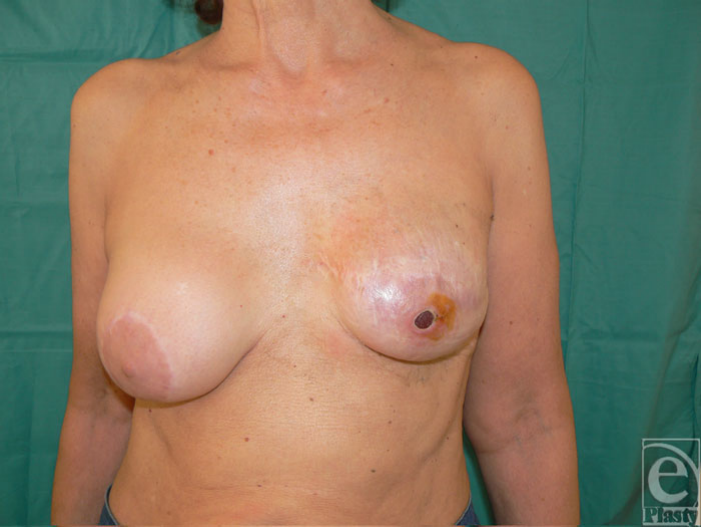
Preoperative view of a 64-year-old woman with breast implant exposure after areola grafting.

A 64-year-old woman underwent right mastectomy, followed by breast reconstruction with expander and permanent implant. Postoperatively, the patient developed a draining fistula over the implant (Fig 11).

**Figure 12:**
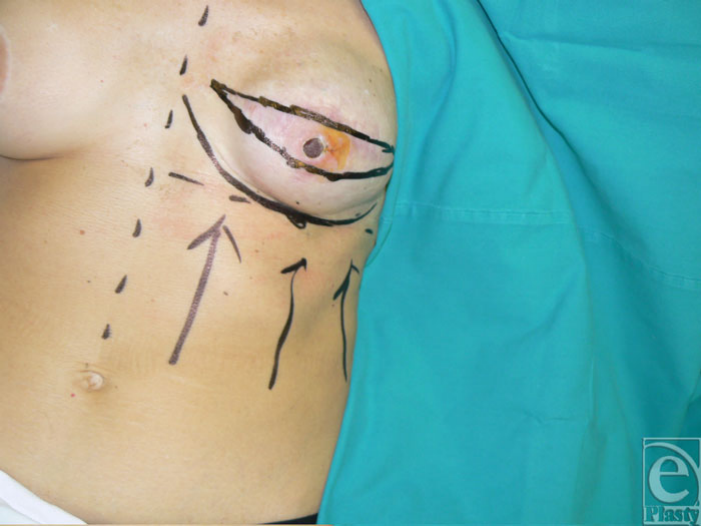
A wide skin excision and reconstruction with abdominal advancement flap.

A 5 × 15-cm area of thin damaged skin was excised and reconstructed with an abdominal advancement flap (Fig 12).

**Figure 13:**
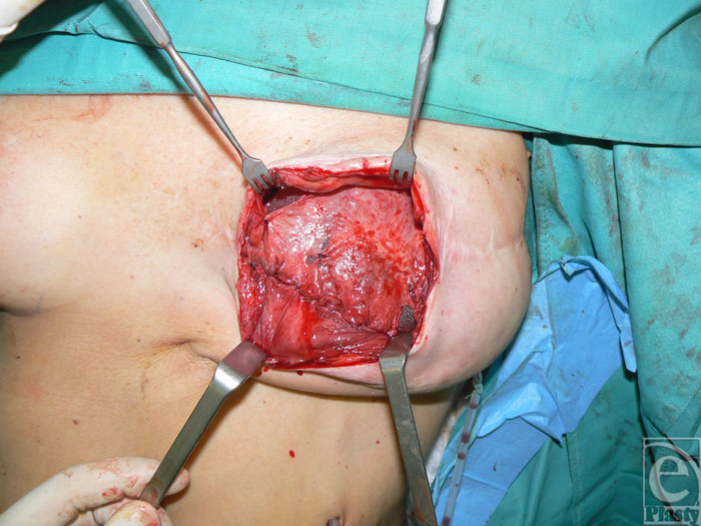
The abdominal flap is advanced, the inframammary fold is reinstated, and the capsular flap harvested from the chest wall is secured to the capsule at the inferior pole.

After recreating the inframammary fold, the soft tissues were reinforced with a superiorly based capsular flap harvested from the chest wall (Fig 13).

**Figure 14:**
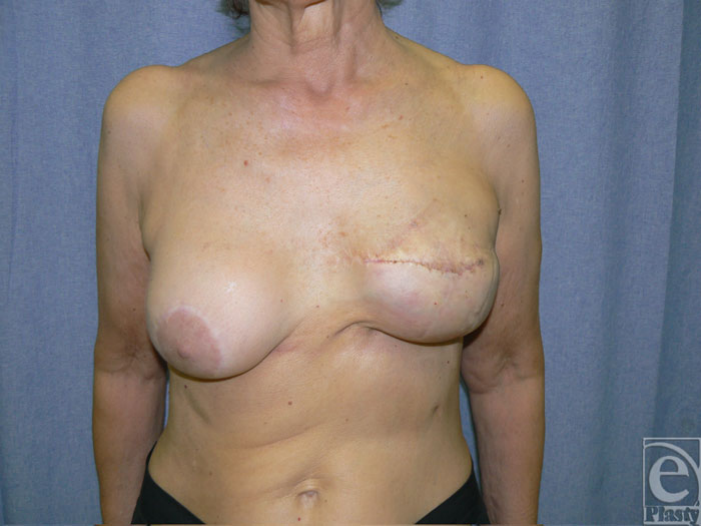
Postoperative result showing the success of the procedure and prevention of breast device exposure.

The final result shows the abdominal advancement flap, recreation of the inframammary fold, and prevention of implant exposure utilizing the capsular flap (Fig 14).

## RESULTS

**Figure 15:**
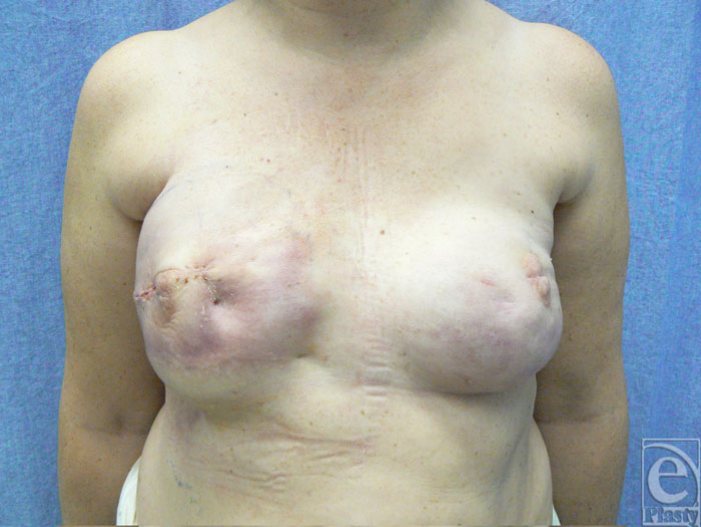
Skin irregularities are present for few weeks after the procedure and will disappear with time. See the same patient after few months in Figure 8.

Complete correction of the extruded implants was achieved with the use of capsular flaps in all patients treated. Temporary skin depressions were seen in the acute phase of healing due, in part, to wound contracture and negative pressure of the suction drains (Fig 15).

Both skin retractions and irregularities disappeared completely at 6 months (Fig 8). No complications, such as implant extrusion, hematoma, seroma, or infection, occurred.

## DISCUSSION

The senior author's interest in this subject started with his 2002 publication^9^,^10^ describing for the first time the innovative use of capsular flaps to prevent palpable wrinkling of implants. Since then, other authors have successfully used this technique to correct implant rippling^11^ or other breast deformities.^12^

Surgical strategies of implant salvage have been described with successful results by renowned surgeons in cases of breast implant extrusion and infection.^1^–^4^ Nevertheless, Spear et al^4^ recognized that poor results can be obtained with conventional methods when deficient soft tissue coverage or overwhelming infection is present. In these challenging cases, implant removal and delayed reconstruction with implants or autologous flaps seem to be the only available option.

Capsular formation around a breast implant is a physiological response to a foreign body,^13^ and it increases when radiation therapy is administered.^7^ The viability of capsular flaps has been demonstrated to be adequate in experimental animal models, in which the flaps have been used as a recipient area for skin grafts,^14^ random flaps,^15^ or axial flaps.^16^,^17^ In this study, vascularized capsular flaps were harvested in unirradiated areas and inset when possible in multiple layers. This allows for the reinforcement of the breast envelopes and prevents recurrence of implant extrusion. The capsular flaps can be harvested either from the anterior breast envelope (cases 1–3) or from the chest wall (case 4). The flap can be superiorly, inferiorly, medially, or laterally based, depending on tissue viability and defect location. The authors advocate the use of this innovative technique as an alternative tool when other conventional methods have failed to avoid implant removal and the need for delayed reconstruction.
